# Using the Behavior Change Technique Taxonomy v1 to conceptualize the clinical content of Breaking Free Online: a computer-assisted therapy program for substance use disorders

**DOI:** 10.1186/s13011-016-0069-y

**Published:** 2016-07-22

**Authors:** Stephanie Dugdale, Jonathan Ward, Jan Hernen, Sarah Elison, Glyn Davies, Daniel Donkor

**Affiliations:** Breaking Free Group, Manchester, UK; Turning Point, London, UK

**Keywords:** Substance use disorders, Behavioral change techniques, Computer-assisted therapy

## Abstract

**Background:**

In recent years, research within the field of health psychology has made significant progress in terms of advancing and standardizing the science of developing, evaluating and reporting complex behavioral change interventions. A major part of this work has involved the development of an evidence-based Behavior Change Technique Taxonomy v1 (BCTTv1), as a means of describing the active components contained within such complex interventions. To date, however, this standardized approach derived from health psychology research has not been applied to the development of complex interventions for the treatment of substance use disorders (SUD). Therefore, this paper uses Breaking Free Online (BFO), a computer-assisted therapy program for SUD, as an example of how the clinical techniques contained within such an intervention might be mapped onto the BCTTv1.

**Method:**

The developers of BFO were able to produce a full list of the clinical techniques contained within BFO. Exploratory mapping of the BCTTv1 onto the clinical content of the BFO program was conducted separately by the authors of the paper. This included the developers of the BFO program and psychology professionals working within the SUD field. These coded techniques were reviewed by the authors and any discrepancies in the coding were discussed between all authors until an agreement was reached.

**Results:**

The BCTTv1 was mapped onto the clinical content of the BFO program. At least one behavioral change technique was found in 12 out of 16 grouping categories within the BCTTv1. A total of 26 out of 93 behavior change techniques were identified across the clinical content of the program.

**Conclusion:**

This exploratory mapping exercise has identified the specific behavior change techniques contained within BFO, and has provided a means of describing these techniques in a standardized way using the BCTTv1 terminology. It has also provided an opportunity for the BCTTv1 mapping process to be reported to the wider SUD treatment community, as it may have real utility in the development and evaluation of other psychosocial and behavioral change interventions within this field.

## Background

In recent years, the discipline of health psychology has made significant progress in terms of advancing the science of behavioral change intervention development [1]. Much of this work has centred on creating an evidence-based taxonomy of all available intervention techniques that might be included in complex interventions. Complex interventions refers to those with multiple interacting components [2]. These interventions are commonly used in healthcare and may facilitate behavioral changes. The primary motivation for this work around taxonomy development centres around calls made for the Consolidated Standards of Reporting Trials (CONSORT) [3, 4], which has highlighted that in randomized clinical trials (RCT), reporting of the content of the ‘active ingredients’ of complex behavior change interventions needs to be more precise.

To date, poor reporting of the active ingredients contained within such interventions has been demonstrated to impede replication of interventions, which can have major implications for the fidelity of delivery [5, 6]. If an intervention is not described properly, this may result in different facilitators delivering the intervention in very different ways, which may impact significantly on the clinical effectiveness and the overall implementation of the intervention [7, 8]. Additionally, difficulties with accurate replication may also impact evaluation, as outcomes from interventions require replication across multiple time points before reliable conclusions around effectiveness can be reached [9, 10]. Other issues with evaluation that may result from poor reporting of intervention content lie in the difficulties of synthesizing findings from across multiple studies in systematic reviews and meta-analyses. If the interventions within studies are not adequately described, it is difficult to know whether the interventions being included within a meta-analysis are equivalent in terms of content [11], as interventions that are very different from one another should not be included in meta-analyses [12, 13].

These difficulties in relation to the reporting of complex behavior change interventions have led to a program of research in which a standardized taxonomy describing specific active ingredients within interventions has been developed, the Behavior Change Technique Taxonomy v1 (BCTTv1), [10, 14]. Behavior change techniques are defined as the smallest observable and replicable components of an intervention which can lead to behavior change [5]. The BCTTv1 identifies 93 behavior change techniques, grouped into 16 categories of change, and has been developed by a consortium of health psychologists and other behavioral change experts using a Delphi exercise to increase the reliability of the taxonomy [5]. The purpose of this exercise was to provide a common language for describing these active components of behavior change interventions to facilitate the effective and precise replication of delivery of such complex multi-component interventions, thereby enhancing fidelity of delivery and facilitating replicability, evaluation and synthesis of evaluation outcomes via meta-analyses [15].

Although the BCTTv1 approach to describing intervention content is becoming more widely used amongst health psychologists and public health specialists in relation to interventions for behaviors such as smoking [16] or physical activity [17], this approach has so far not been translated into standard intervention development and evaluation within the SUD field. Therefore, the lack of a standardized means of describing the clinical content of interventions within the SUD sector means that many reviews of the literature have struggled to synthesize, via meta-analyses, the evidence from multiple outcomes evaluations from these interventions in order to ascertain which are most effective. For example, in a systematic review of the literature exploring treatment for SUD and co-occurring mental health difficulties, Drake and colleagues [18] discussed their difficulties synthesizing the literature due to differences in terminology used to describe intervention structure and content across similar treatment options, and called for increased standardization to overcome this. Especially within systematic Cochrane reviews, this lack of consistency and transparency in describing clinical content across complex interventions can be problematic. This results in potentially insufficient detail around the clinical content of many studies, which can prevent reliable syntheses of findings in systematic reviews via meta-analyses [19–22]. Given the broad scope of SUD behavioral change interventions reported in the literature, providing a standardized taxonomy such as the BCTTv1 to describe these intervention components may help to create a common clinical language, and consequently facilitate fidelity of delivery (for example, by developing a ‘common language’ coding schedule for evaluation of fidelity), replication, evaluation and synthesis of findings from outcomes evaluations.

In an effort to introduce the SUD field to the BCTTv1, and develop a common language for the precise reporting of intervention content, in line with recommendations proposed by CONSORT [3, 4], Breaking Free Online (BFO) is presented as an example SUD psychosocial intervention onto which it may be possible to map techniques from the BCTTv1. BFO is a structured intervention program for SUDs, which is delivered on a digital platform as computer-assisted therapy (CAT) and is intended for anyone attempting to overcome problem use, habitual use, dependence or addiction, and also for those trying to maintain abstinence. The use of this digital platform via CAT also ensures treatment fidelity is optimized, as intervention techniques are delivered by a computer in a highly standardized manner [23, 24], without the human-related variance in delivery often seen in more traditional group or other human-facilitated interventions [8, 25]. Therefore, CAT interventions are not as subject to the effects of compromised fidelity from treatment, which may impact on clinical effectiveness [26]. CATs have already demonstrated efficacy in the treatment of substance misuse difficulties, for example other implemented interventions CBT4CBT available in the US [27] and SHADE available in Australia [28] have reported favourable outcomes in the reduction of substance use and co-morbid symptoms of depression [29–31]. In contrast, BFO is also appropriate for those individuals with a ‘dual-diagnosis’ of co-morbid SUD and mental health difficulties, including both anxiety and depression, and has been found to be effective for a range of SUD populations in reducing their substance use and dependencies, and improving their mental health and quality of life [32–36]. The program is currently commissioned and being delivered by SUD treatment and recovery services across the UK, enabling service users to access the program free of charge. However, after initial access of the program through services, service users may also access the program at home via the internet.

**Fig. 1:**
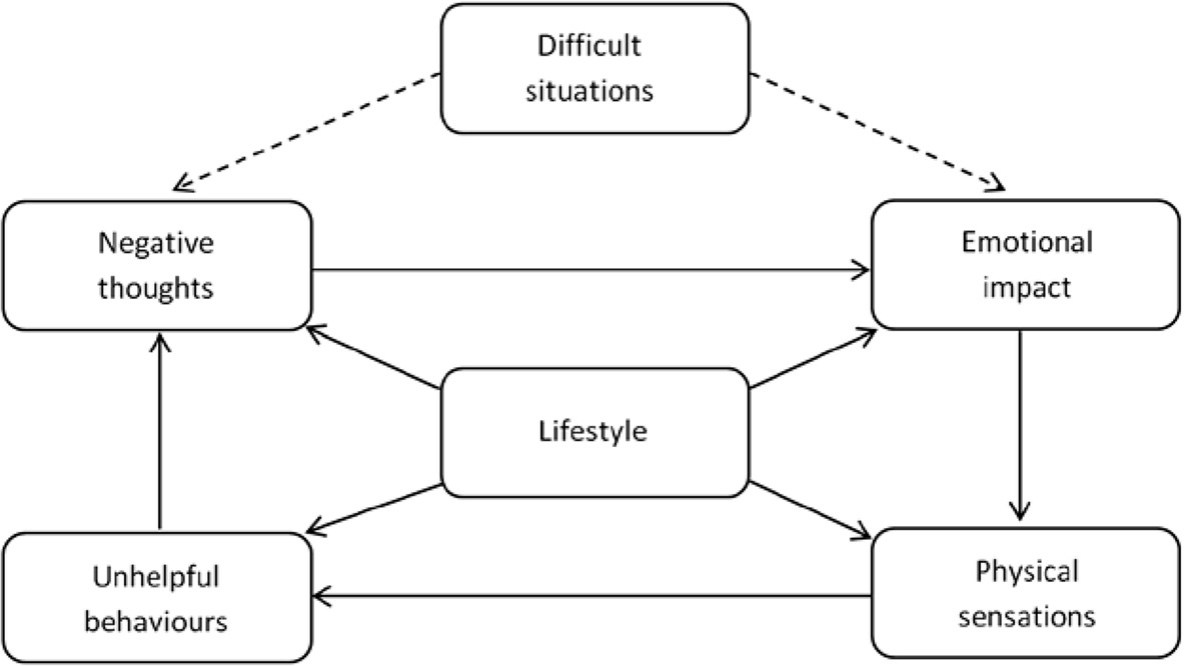
Lifestyle balance model

The use of structured evidence-based psychosocial interventions such as this are increasingly being demonstrated to be an effective means of addressing substance use disorders (SUD). Programs such as the UK International Treatment Effectiveness Project (ITEP) [37, 38] are showing the utility of taking a systematic approach to intervention development. This approach involves identifying areas of psychosocial difficulty that individuals with SUD might be experiencing and then applying appropriate evidence-based interventions to address these. In line with good practice guidelines published by the British Psychological Society [39], a standard clinical case formulation approach was taken when developing BFO, which involved a systematic process comprised of a series of steps. Firstly, the common underlying biopsychosocial difficulties that are implicated in SUD, and how these areas of difficulty might relate to one another, were examined by drawing on SUD theory and evidence in the literature. These areas of difficulty were conceptualized via a biopsychosocial domain model, the ‘Lifestyle Balance Model’ (LBM: [40]), which was developed by the authors and is derived from the standard five-factor cognitive behavioral therapy (CBT) model [41, 42], with the additional central component of lifestyle (see Fig. 1). The LBM comprises six domains of biopsychosocial functioning that are implicated in SUD and recovery: lifestyle, negative thoughts, emotional impact, unhelpful behaviors, difficult situations and physical sensations. See Davies *and colleagues* [40] for a full discussion of the development of the LBM and the evidence-base underpinning it.

Following this phase, the literature was searched again in order to identify evidence-based intervention techniques appropriate for addressing the areas of biopsychosocial difficulty contained within the LBM. Assigned to the six domains of the LBM, these interventions provide a combination of psychoeducation and more practical, action-oriented techniques that support the user to achieve enduring cognitive and behavioral change in these six areas of biopsychosocial functioning. Once intervention techniques were identified, consultation was carried out with stakeholders within the SUD sector in order to make refinements to the BFO program. This included meeting with professionals who commission, manage and work within specialist SUD services, and also individuals receiving treatment for SUD, to generate detailed feedback on the BFO program content. The process of BFO development described above reflects the professional disciplines of the principal creators of the program: a Clinical Psychologist with extensive experience of clinical assessment and case formulation and the delivery of cognitive behavioral interventions in clinical practice with adult clients, and the development of training programs for frontline practitioners in utilizing cognitive behavioral therapeutic techniques (JW); and a substance misuse commissioner, manager and practitioner (GD) who had previously utilized approaches such as those outlined by ITEP [37, 38].

The approach taken to the development of BFO may be in keeping with the clinical expertise of the developers of the program, however, with recent advances in health psychology around the development of the BCTTv1, the application of this taxonomy may be important in enabling the accurate reporting of the techniques involved within this intervention. This may further increase evaluation and implementation of BFO, through the use of a ‘common language’ applicable across different services and sectors in describing this intervention. This paper therefore attempts to introduce the SUD field to the BCTTv1 in order to contribute to ongoing efforts to develop more effective interventions, and to more accurately and systematically describe the clinical content within these interventions. This aim of this paper is furthered by using the example of BFO, primarily through describing its clinical content using the BCTTv1. This may support developers of SUD interventions to increase efficacy and report more active treatment ingredients clearly. This contributes to aims proposed by CONSORT in more precise reporting of complex intervention content.

## Methods

This exploratory paper investigates the potential of the BCTTv1 to aid description of the clinical content contained within BFO. To achieve this, the clinical content of BFO was analysed and relevant behavior change techniques that accurately captured and described the individual strategies contained within the programme were mapped onto the BCTTv1. This process is now discussed.

The developers of BFO were able to produce a full list of the clinical techniques contained within BFO. Initial coding of the behavior change techniques within BFO was conducted by SD and SE, along with JW, a clinical psychologist who led on the development of BFO, using the list of clinical techniques within BFO. All authors have received online training around the coding of behavior change techniques from the BCTTv1 (www.bct-taxonomy.com). Each BFO technique was described both in terms of SUD terminology and through referencing relevant techniques from the BCTTv1 [5]. This process was carried out alongside the development of the latest version of the BFO program (V3). The coded mapping of BFO to BCTTv1 techniques produced by each of the three authors were then combined into one coded list, through discussing individual findings and resolving any discrepancies. If any discrepancies arose between the authors during coding, this was resolved by presenting a justification for why they believed that a behavior change technique was appropriate, and discussed until an agreement was reached. The coded techniques from the BCTTv1 which corresponded with the relevant intervention techniques and targets within BFO were then disseminated to another of the developers of BFO (GD) and two clinical psychologists with experience in the clinical application of behavior change techniques (JH and DD). The coded techniques were reviewed and any discrepancies in the coding were discussed between all authors until an agreement was reached. This aim of this was to ensure that the findings fit with initial intervention consultation that occurred during development of the program.

## Results

The findings from the exploratory coding exercise are now discussed. Overall, of the 16 categories within the BCTTv1, 12 of these contained behavior change technique that mapped onto the techniques contained within BFO. No BFO techniques were found to relate to the BCTTv1 categories of ‘comparison of behavior’, ‘associations’, ‘comparison of outcomes’, or ‘covert learning’. See Table 1 for a reference of which of the 16 categories within the BCTTv1 relate to specific techniques within the BFO program. A total of 26 out of 93 behavior change techniques from the BCTTv1 were identified within the BFO program. See Table 2 for a summary of all the information provided within this section. The findings of this exploratory mapping exercise are now discussed. As the content of BFO is derived from cognitive behavioral theory, the specific intervention targets described here have been divided between those that address cognitive, emotional or behavioral factors, or a combination of these, in order to demonstrate how these factors link to the clinical structure of the program.

**Table 1:** Categories of behaviour change techniques identified within BFO

	Techniques contained within BFO														
BCTTv1 categories	Node-link mapping/psychoeducation	Motivational enhancement	Implementation intentions	Cognitive restructuring/mind traps	Reward and reinforcement	Refusal and assertiveness skills	Harm reduction	Activity scheduling	Self-monitoring	Emotional regulation	Coping strategy enhancement	Relapse prevention	Crisis management	Goal setting	Mindfulness-based cognitive therapy
1) Goals and planning	-	-	X	-	-	X	-	X	-	-	X	X	-	X	-
2) Feedback and monitoring	-	-	-	-	-	-	-	-	X	-	-	-	-	-	-
3) Social support	-	-	-	-	-	-	-	-	-	-	X	-	X	-	-
4) Shaping knowledge	X	-	-	X	-	X	-	-	-	-	-	-	-	-	X
5) Natural consequences	X	-	-	-	-	-	X	-	-	X	-	-	-	-	-
6) Comparison of behaviour	-	-	-	-	-	-	-	-	-	-	-	-	-	-	-
7) Associations	-	-	-	-	-	-	-	-	-	-	-	-	-	-	-
8) Repetition and substitution	-	-	-	-	-	X	X	-	-	X	X	-	-	-	X
9) Comparison of outcomes	-	-	-	-	-	-	-	-	-	-	-	-	-	-	-
10) Reward and threat	-	X	-	-	X	-	-	-	-	-	-	-	-	-	-
11) Regulation	-	-	-	-	-	-	-	-	-	X	-	-	X	-	X
12) Antecedents	-	-	-	-	-	X	-	-	-	-	X	-	-	-	-
13) Identity	-	-	-	X	-	-	-	-	-	-	-	-	-	-	-
14) Scheduled consequences	-	-	-	-	X	-	-	-	-	-	-	-	-	-	-
15) Self-belief	-	X	-	-	-	-	-	-	-	-	-	-	-	-	-
16) Covert learning	-	-	-	-	-	-	-	-	-	-	-	-	-	-	-

**Table 2:** The behavioral change techniques contained within Breaking Free Online using the taxonomy produced by Michie et al. [5]

Techniques within BFO (area of LBM/program)	Intervention target	BCT taxonomy (number in taxonomy)
Cognitive		
Node-link mapping/psychoeducation (all LBM)	Case formulation – understand the links between situations, thoughts, emotions, behaviors, physical sensations and lifestyle	Information about antecedents (4.2); Information about health consequences (5.1); Salience of consequences (5.2); Information about social and environmental consequences (5.3); Information about emotional consequences (5.6)
Motivational enhancement (lifestyle)	Increase treatment engagement and retention. Increase readiness to change behavior	Non-specific reward (10.3); Focus on past success (15.3)
Implementation intentions (lifestyle)	Situational cue to elicit behavior	Action planning (1.4)
Cognitive restructuring/mind traps (negative thoughts)	Challenge thoughts that may be unhelpful and linked to situations in which substances might be used	Re-attribution (4.3); Framing-reframing (13.2)
Behavioral		
Reward and reinforcement (achievements)	Encourage new behavior and continuation of this behavior via positive feedback	Non-specific reward (10.3); Non-specific incentive (10.6); Reward approximation (14.4); Rewarding completion (14.5);
Refusal and assertiveness skills (difficult situations)	Relapse prevention for coping with environmental/situational/emotional triggers	Problem solving (1.2); Action planning (1.4); Instruction on how to perform a behavior (4.1); Behavioral practice/rehearsal (8.1); Behavior substitution (8.2); Avoidance/reducing exposure to cues for the behavior (12.3)
Harm reduction (information on alcohol and drugs)	Reduce negative or fatal consequences of substance using behaviors	Information about health consequences (5.1); Salience of consequences (5.2); Behavior substitution (8.2); Habit reversal (8.4)
Activity scheduling (unhelpful behaviors)	Increase activity to increase energy levels and relieve boredom	Goal setting (behavior) (1.1); Action planning (1.4)
Self-monitoring (progress check)	Monitor behavior to provide feedback about progress towards goals	Self-monitoring of behavior (2.3); Feedback on outcome(s) of behavior (2.7)
Affective		
Emotional regulation (understanding your emotions)	Recognize/understand/normalize emotions	Information about emotional consequences (5.6); Behavioral practice/rehearsal (8.1); Reduce negative emotions (11.2)
Coping strategy enhancement (understanding your emotions)	Developing more appropriate coping strategies	Problem solving (1.2); Social support (unspecified) (3.1); Behavioral practice/rehearsal (8.1); Distraction (12.4)
Cognitive-behavioral		
Relapse prevention (understanding your difficult situations)	Creating action plans on how to avoid or cope in high risk situations	Goal setting (behavior) (1.1); Problem solving (1.2); Action planning (1.4)
Crisis management (understanding your difficult situations)	Intervention to help people in distress access support	Social support (unspecified) (3.1); Reduce negative emotions (11.2)
Goal setting (lifestyle)	Creating SMART goals for recovery	Goal setting (behavior) (1.1); Problem solving (1.2); Goal setting (outcome) (1.3); Action planning (1.4)
Cognitive-behavioral-affective		
Mindfulness-based cognitive therapy (understanding your emotions)	Relapse prevention based techniques	Instruction on how to perform a behavior (4.1); Behavioral practice/rehearsal (8.1); Reduce negative emotions (11.2)

### Intervention techniques in BFO targeting cognitive factors

#### Node-link mapping

Node-link mapping is an intervention technique in which various aspects of functioning are visually represented. Node-link mapping provides the structure for BFO content based upon the theoretical model underpinning the program, the Lifestyle Balance Model (LBM: [40]). Node-link maps allow the individual to visualize how different aspects of their lives and functioning inter-connect. This shares some commonalities with case formulation approaches often used in CBT [41, 43]. Throughout the SUD sector, node-link mapping has been found to be an effective tool for those who experience co-morbid substance use and mental health difficulties. In particular, those with psychological difficulties reported more positive perceptions of health keyworkers when using node-link mapping in comparison to those who only received treatment as usual [44]. Further evidence suggests that participants who used node-link mapping techniques in addition to receiving treatment as usual reported significantly lower rates of opiate use compared to those who only received treatment as usual [45]. This approach also underpins ITEP as node-link mapping provides a simplified, structured and effective method to the delivery of psychosocial interventions, and has demonstrated increased patient satisfaction [46]. The LBM is presented in the format of a node-link map to complement treatment provision in line with the ITEP agenda endorsed by Public Health England.

Behavior change techniques which link to node-link mapping are information about antecedents, information about health consequences, salience of consequences, information about social and environmental consequences, and information about emotional consequences [5]. Node-link mapping in BFO uses psychoeducation as a means to conceptualize this process, therefore, the behavior change techniques contained within node-link mapping may also be contained within the technique of psychoeducation.

#### Psychoeducation

Psychoeducation consists of information about the links between various aetiological factors underpinning a given morbidity, in this case SUDs. Such factors may include environmental, cognitive, emotional, behavioral and lifestyle contributors to and consequences of SUDs and the ways in which these factors may be associated with clinical outcomes. Providing psychoeducation about the links between cognitive, emotional and behavioral factors has been demonstrated to be effective in reducing drinking frequency in service users dependent on alcohol [47], although research has demonstrated that participants who only receive psychoeducation reported significantly higher rates of drug use following intervention compared to those who received CBT [48]. However, this study also found that those who received psychoeducation still reported a significant reduction in drug use after the intervention compared with prior to receiving any support. This suggests that, despite its comparative weaknesses as a stand-alone intervention compared with CBT, psychoeducation is still a viable and clinically effective intervention for SUDs. The potential for using psychoeducation in conjunction with CBT techniques may also overcome any weaknesses of these therapies as stand-alone interventions. BFO utilizes psychoeducational resources alongside other psychosocial techniques, including CBT, to help service users understand their difficulties and the importance of taking positive action to address identified areas of difficulty within the LBM.

Components of the BCTTv1 which link to psychoeducation are information about antecedents, information about health consequences, salience of consequences, information about social and environmental consequences, and information about emotional consequences [5]. The relevant behavior change techniques contained within psychoeducation also inform node-link mapping and the personalized content contained within the nodes.

#### Motivational enhancement

Enhancing motivation to change is beneficial for SUDs as it increases treatment engagement and retention and can therefore improve outcomes [49]. Research has demonstrated that use of motivational enhancement therapy (MET) can lead to lower blood alcohol concentration [50], as well as reducing drinking frequency in service users with alcohol dependency and improving their general functioning, particularly social functioning [51]. This can be achieved through enabling the individual to explore or recognize a problem and helping them to develop an action plan to overcome this [52].

Rohsenow and colleagues [53] found that in 165 cocaine dependent service users with particularly low initial motivation to engage in treatment, MET resulted in decreased substance use and a reduced rate of relapse at one year follow-up. However, it was also found that those with initially high motivation did not reduce their substance use intake at follow-up after engaging in motivational enhancement, potentially due to these techniques being perceived as unnecessary if the service user was already motivated to change their behavior, although this effect size was only small (ƒ = 0.20) and restricted to an in-patient participant sample. Participants after an MET intervention viewed cocaine as having stronger negative effects. This further reinforces the benefit of this approach in increasing levels of engagement with therapy and in altering perceptions of substance use. The BFO intervention uses MET to provide continual feedback to the service user regarding their recovery progression through a range of visual devices depicting their substance use, emotional wellbeing, resilience and quality of life. This links to the behavior change technique of focusing on past successes and providing a non-specific reward [5].

#### Implementation intentions

Implementation intentions connect an intention to behave in a specified manner within a particular context, thus creating a situation-specific cue to perform the behavior [54]. Implementation intentions are typically represented as ‘IF…THEN’ statements, which describe a potential situation that could occur (for example ‘IF I am near the bar’) and then specify a behavior which will be enacted (‘THEN I will phone my support worker’). Evidence suggests that those who formed implementation intentions regarding their alcohol use reported reduced rates of drinking at follow-up compared to controls, although the strength of the implementation intention is dependent upon the level of commitment to this from the participant [54]. This is important to consider when developing plans as it may affect enactment of behavior; however it could be seen that approaches such as motivational enhancement, which increase engagement, may help to increase the strength of commitment. Implementation intentions link to the behavior change technique of action planning [5] and BFO uses this strategy within goal setting to increase the probability of goal attainment.

#### Mind traps and cognitive restructuring

Cognitive distortions or thinking errors are called ‘mind traps’ within the application of ITEP. This is where people experience a particular thinking style that may be subjectively biased or distorted, which can trigger substance use. Those who experience a mental health problem such as depression can be thought to have an automatic negative bias about themselves, the world and their future [55]. Cognitive restructuring or reframing teaches people to review such negative thoughts, and mind traps, to see whether or not there is an alternative way to view the situation and to challenge beliefs [41, 56]. For example, a service user may believe they are helpless in a particular situation and that using a substance will help them with a challenging aspect of life without acknowledging the negative consequences of their drug use [57].

Cognitive restructuring is central to CBT [58, 59], and has been demonstrated to be an integral part of CBT-based relapse prevention approaches for SUDs [58]. Within BFO, service users learn to identify when they experience specific mind traps. These include the ‘helpless trap’ (as explained above), the ‘blame trap’ (where they do not accept personal responsibility for their actions, including those related to or arising from their substance use), the ‘guilt trap’ (where substances can be used to mask feelings of remorse), the ‘catastrophe trap’ (where everything is blown out of proportion and substances can be used as a coping method), and the ‘all or nothing trap’ (which may render a service user who experiences a slip more likely to succumb to a full blown lapse). Cognitive restructuring would encourage them objectively to evaluate this decision before acting upon it. The strategy of testing out thoughts and beliefs can be linked to re-attribution and re-framing in the BCTTv1 [5].

### Intervention techniques in BFO targeting behavioral factors

#### Reward and reinforcement

Reward and reinforcement involves providing a tangible reward or some other form of positive feedback such as praise for the enactment of a desired behavior, in order to reinforce the behavior [5]. Research has found that cocaine and heroin users are more likely to secure employment and abstain from substance use following treatment if their behavior is rewarded, compared to those who do not receive a reward for their behavior [60–62]. The use of rewards can also reinforce participation in recreational non-drug related activities [63]. However, the use of rewards to reinforce positive outcomes is directly proportional to the immediacy and value of the reward: the greater and more imminent the reward, the stronger the likelihood of the desired outcome [64]. Therefore it is important to determine and deliver the appropriate level of reward for a behavior, in order to achieve the maximum desired outcome. The removal of the reward should also be considered, for example if the reward stops then behavior may also stop. However evidence from Petry and colleagues [65] has suggested that after the removal of the reward, behavioral changes can still be maintained, in this instance with regard to drug abstinence and quality of life. The BFO intervention program utilizes this technique by providing service users with visual rewards such as trophies, rosettes, medals and garlands for attaining different levels of progress in relation to their recovery. BFO users also receive personalized certificates for their achievements, which they can download and print. The behavior change techniques which relate to reward and reinforcement are non-specific reward, non-specific incentive, reward approximation and reward completion [5].

#### Refusal and assertiveness skills

Refusal and assertiveness skills demonstrate how to respond assertively to people, as opposed to reacting aggressively or passively. These skills have been demonstrated to be effective in teaching those with alcohol dependence an appropriate way to refuse the offer of a drink [66, 67]. Within the BFO program, this technique is part of a broader relapse prevention strategy referred to as ‘recognize-avoid-cope’. Within this strategy, the components ‘recognize’ and ‘avoid’ are specifically related to refusal and assertiveness skills, referring to the service user’s ability to recognize a high-risk situation and take proactive steps to avoid that situation if at all possible. Previous research has found that the ability to refuse substances, and to be assertive when doing this, was significantly lower in adolescents and males compared to their counterparts, thereby increasing the likelihood of substance using behavior [68–70]. Therefore it is important that these skills are explored and developed, particularly within this population. Assertiveness and refusal skills are linked to the behavior change techniques of problem solving, action planning, instruction on how to perform a behavior, behavioral practice, behavior substitution, and reducing exposure to cues for the behavior [5].

#### Harm reduction

Harm reduction techniques aim to reduce negative consequences such as injury and death associated with substance using behavior. Work by Marlatt and Witkiewitz [71] found evidence to support the efficacy of harm reduction techniques in reducing alcohol use and related negative consequences compared to standard abstinence-based programs. Harm reduction principles can be useful in informing intervention approaches that address a range of high-risk behaviors such as substance use [72] and behaviors associated with this such as injecting substances [73]. Whilst this technique implies that the behavior may not necessarily cease, it provides individuals who may not be ready, or do not wish to pursue abstinence, with the knowledge required to use substances more safely. Within BFO, drug awareness and overdose management information resources are provided that ensure service users have access to accurate and factual information. This links to the behavior change techniques of information about health consequences, salience of consequences, behavioral substitution and habit reversal [5].

#### Activity scheduling/behavioral activation

Activity scheduling is a technique commonly used in CBT to promote wellbeing and overcome the reduction in behavioral activity typically associated with periods of poor mental health, particularly depression [41]. Low mood is prevalent during detoxification and in the early stages of recovery. Included in this reduction of activity is a reduction in behaviors that are found to be pleasurable and those that lead to a sense of achievement and self-esteem [42], which may contribute to substance use as a form of coping. Behavioral activation also teaches service users to build routine, avoid boredom and begin to restore structure and a sense of normality to their daily life, which may often be negatively influenced by substance use. This links to goal setting (behavior) and action planning in the BCTTv1 [5].

Studies have demonstrated that setting structured goals for enactment of activities for achievement and self-esteem can significantly improve mood [74]. Activity scheduling has also been shown to be effective in supporting drug-dependent individuals in reducing their substance use [75]. In particular, those who engage in increased amounts of leisure activity have been found to report reduced drug use [76]. These benefits could be realized through activity scheduling as an alternative when substances are often used, developing peer or family relations with non-substance users, and increasing life satisfaction by participating in a non-substance using activity that is of interest, including treatment [77].

#### Self-monitoring

Self-monitoring of behavior is in itself contained within the BCTTv1 [5] and involves reflecting upon one’s own thoughts, emotions and behaviors, and keeping a record of these for a specified period of time [78]. Feedback is sometimes also provided in order to demonstrate changes over time in the thoughts, emotions and behaviors being monitored, relating to the behavior change technique of feedback on outcomes of behavior [5]. Self-report has been stated to be an effective method by which to monitor progress in interventions [79], with self-reports suggested to be more honest and reliable when carried out on a computer due to the confidential nature of this method of assessment [80]. Self-monitoring has been identified as a key behavioral change technique for reducing excessive alcohol consumption [81], and also for addressing other forms of SUD, especially when implemented using digital technologies [82]. However, there are factors which can affect the validity of the self-report method. These may include forgetting, memory distortion or the social context within which the self-report occurs, for example within a treatment service, with a practitioner or at home [83]. However self-report methods have typically been found to be reliable, and the use of computers to record this data can also help to minimize errors [84]. Throughout the BFO intervention, service users are required to self-monitor their substance use and key ingredients of recovery progression such as emotional, wellbeing, resilience and quality of life.

### Intervention techniques in BFO targeting affective factors

#### Emotional regulation

Emotional regulation incorporates learning how to recognize, understand and normalize emotions, and to avoid ignoring or supressing feelings [85]. This is particularly pertinent as evidence indicates common use of substances as a coping mechanism and also as a method of increasing positive emotional states [86]. The literature suggests that impaired substance use-related control is associated with poor emotion regulation [87]. Similarly, increased emotion regulation skills have been found to decrease substance use, particularly in those with dually diagnosed mental health difficulties [88]. In order to facilitate this change in the understanding of how emotions and substance use interrelate, BFO provides psychoeducation about the links between thoughts, emotions, behaviors and substance use, and how to recognize the early warning signs which could signal poor emotional wellbeing triggering lapse or relapse. Emotion regulation links to the behavior change techniques of information about emotional consequences, behavioral practice and reducing negative emotions [5].

#### Coping strategy enhancement

Poor coping techniques are associated with SUDs [56] and so coping strategy enhancement is used to develop coping techniques that are more appropriate and effective than using substances [89–91]. This links into the previous discussion of emotion regulation, which suggests that substance use may in itself serve as a coping mechanism for managing negative emotions [86]. The use of effective coping strategies in a risky situation has been found to lead to an increase in self-efficacy (belief in one’s own ability), resulting in a decreased likelihood of relapse [92]. Conversely, this could mean that ineffective coping strategies can ultimately contribute to relapse. Suggested effective coping strategies include reinforcement techniques and relapse prevention [93]. In BFO, techniques such as ‘attention switching’ and ‘attention narrowing’ are also used, linking in to the behavior change technique of distraction [5]. The service user is guided through a virtual walk through a forest (in the form of an immersive audio-visual sequence) and prompted to attend to all their senses [94], in order to shift their attention away from symptoms of heightened physiological arousal which can be caused by, for example, cravings for substances or affective states such as anxiety or anger. Distraction has been evidenced as being more effective than thought suppression, which has been linked with an actual increase in the unwanted thought [95]. Coping strategy enhancement uses the behavior change techniques of problem solving, social support (unspecified), behavioral practice and distraction [5].

### Intervention techniques in BFO targeting cognitive-behavioral factors

#### Relapse prevention

Work by McLellan and colleagues [96] indicates that SUDs are chronic relapsing conditions. Therefore it is imperative that relapse prevention strategies are available for those in treatment. Techniques used in BFO to prevent relapse include understanding the consequences of drug use and creating action plans to avoid lapse or relapse in risky situations [5, 56, 91]. These approaches have been reported to have efficacious outcomes in reducing the severity of relapse and increasing the durability of relapse prevention, regardless of substance type [97]. Further evidence suggests that although relapse prevention may not be as effective as active interventions used for SUDs, it is still thought to be a reliable and effective intervention method [98]. Relapse prevention is the main focus of the ‘cope’ component of the ‘recognize-avoid-cope’ technique used within BFO, which teaches service users to plan ahead to identify high risk situations, work out how to avoid them, and cope positively with them if, for any reason, they cannot be avoided. This relates to goal setting (behavior), problem solving and action planning from the BCTTv1 [5].

#### Crisis management

A crisis can be defined as a stressful life event which poses a challenge or threat and is difficult to cope with [99]. Crisis management acts as an effective short term, fast-acting intervention to support people experiencing distress [100]. Typical stages of crisis management include immediate intervention, stabilising the service user and building up a support network, promoting understanding, problem solving, and increasing self-reliance and self-efficacy [101, 102]. However, crisis management techniques are not meant to be used as a stand-alone therapy and should be used in conjunction with other approaches [103]. The BFO program signposts service users to mutual aid groups – specifically, Alcoholics Anonymous, Narcotics Anonymous, Cocaine Anonymous and SMART Recovery – to help them to identify and access appropriate support networks during times of crisis. Therefore, social support (unspecified) and reducing negative emotions from the BCTTv1 are used in this technique [5].

#### Goal setting

Setting goals related to reducing substance use is important as it provides a focus for recovery [67, 104]. Evidence suggests that effective goals should be specific, measurable, achievable, realistic and time-bound [105]. This is related to the behavior change techniques of goal setting (behavior) and goal setting (outcome) [5]. In the BFO program, goal setting is facilitated within the ‘lifestyle’ component of the LBM. Service users are asked to specify an overall lifestyle goal and then work to achieve this by progressing through a series of systematically planned smaller goals. Service users are also guided to anticipate the barriers they may face when attempting to complete these goals, and plan to overcome these. This process employs the additional behavior change techniques of problem solving and action planning [5].

### Intervention techniques targeting cognitive-behavioral-affective factors

#### Mindfulness-based CBT

Mindfulness is a meditative technique that involves observation and awareness of one’s thoughts and emotions [106]. The integration of mindfulness-based stress reduction [107] and CBT [108] has led to the emergence of mindfulness-based cognitive behavioral therapy, primarily for the treatment of depression. In addition, mindfulness-based CBT has been demonstrated to be effective in reducing the consumption and cravings of substances [109], including reductions to instances of lapse and relapse in SUDs [56, 110, 111]. This approach has also been demonstrated to be effective in the treatment of co-existing mental health and substance misuse issues. In one study, it was found that the relationship between craving and depression was reduced in those practising mindfulness compared to the treatment as usual group [112]. Research has found that participants who completed a mindfulness course demonstrated decreased substance use and cravings, and increases in facets of mindfulness including acting with awareness, compared to the group who participated in treatment as usual [113]. Practising mindfulness strategies, particularly developing an awareness and openness to one’s thoughts and related emotions, as opposed to suppressing them, has been found to benefit emotion regulation [114]. Mindfulness strategies employed within BFO include ‘urge surfing’, which targets cravings for substances, and in the context of ‘attention narrowing’ or focused attention, which is employed as a technique for managing heightened states of physiological arousal. The behavior change strategies used within this technique are instruction on how to perform a behavior, behavioral practice, reducing negative emotions and distraction [5].

## Discussion

Work by CONSORT [3, 4] has led to guidelines outlining how the clinical content, or ‘active ingredients’, of complex behavior change interventions should be more precisely reported in evaluations such as RCTs, in order to allow greater fidelity of implementation when interventions are replicated, and more accurate synthesis of findings across multiple studies in meta-analyses. To facilitate more precise reporting of intervention content, the BCTTv1 may be used to provide a common terminology for the active ingredients contained within interventions [5, 9, 14]. The BCTTv1 has been used in recent years to describe the clinical content of a range of interventions addressing multiple health behaviors. In the SUD treatment field, the BCTTv1 has hitherto not been used to facilitate more precise description of complex intervention elements. Therefore, to demonstrate to the SUD sector the potential for the BCTTv1 in relation to describing SUD interventions, behavior change techniques were mapped onto the psychosocial and behavioral change techniques contained within a structured, psychosocial and behavioral change intervention for SUD, Breaking Free Online (BFO).

This exploratory study mapped the BCTTv1 onto the clinical content of the BFO program. The techniques utilized within the program were decided upon through knowledge of the substance use literature and consultation with experts in the field, as these techniques were identified as being implicated in substance misuse and recovery. Of the 16 categories contained within the BCTTv1, 12 of these contained behavior change techniques that mapped onto the techniques within BFO. The BCTTv1 categories of ‘comparison of behavior’, ‘associations’, ‘comparison of outcomes’, or ‘covert learning’ did not contain any behavior change techniques which mapped onto the content of the program. The process by which the clinical content of BFO was decided ensures that superfluous techniques, which do little to act upon the intervention targets identified, are not included within the program. It may be of benefit for similar interventions to publish the clinical content of their program or the behavior change techniques contained within this. As it stands, few studies publish this information, however dissemination of this evidence may enable comparisons across interventions. Research has already demonstrated the potential for comparisons of behavior change techniques across interventions, for example those related to smoking [115], and diabetes care [116]. The benefits of this approach may extend to further evaluative studies and intervention design in investigating the key components of behavior change specific to substance misuse for example. This may also further develop training competencies for those working in the field, to ensure that they have an understanding of the key techniques required to enable behavior change [115].

Although it was possible to map the taxonomy onto the clinical techniques within BFO, there were some limitations to this approach. Firstly, it was clear through conducting this mapping process that there is a large overlap between some of the techniques and the behavior change techniques ascribed to these. For example, different areas of the LBM including ‘lifestyle’, ‘difficult situations’ and ‘unhelpful behaviors’ all address separate areas of psychosocial functioning, however they all employ ‘action planning’, potentially reflecting the complex nature of some of the techniques within the intervention. On the other hand, the ability to break down this terminology (different psychosocial techniques) into manageable and clearly defined actions (using the BCTTv1) for service use, further demonstrates the need for a standardized terminology in the substance use sector, not least to help other researchers design, replicate and evaluate interventions.

Secondly, the program was designed initially through a clinical case formulation approach, which meant that this exploratory mapping of the BCTTv1 onto the BFO program intervention targets was conducted in a retrospective manner: by identifying the behavior change techniques closely aligned with the clinical techniques that had already been selected for inclusion in the program during the clinical case formulation process. This meant that the BCTTv1 mapping process was open to subjectivity and not intuitive, compared to selecting appropriate behavior change techniques during the initial design when intervention targets were first identified. In order to overcome this subjectivity and increase the inter-rater reliability of this approach, a Delphi study is currently being conducted with professionals working in the field of SUD treatment, to map the BCTTv1 onto the psychosocial techniques contained within the BFO program.

## Conclusion

This study has provided an initial demonstration of the potential for using the BCTTv1 as a standardized terminology when describing clinical content of interventions within the SUD treatment sector, using the clinical content of BFO as an example. Implications from this research may extend to healthcare professionals or researchers, to encourage the use of clinically effective psychosocial interventions within the substance use sector, and provide a basis for the development of future psychosocial interventions and the effective evaluation, replication and synthesis of current interventions for SUD.

## Abbreviations

BCTTv1, Behavior Change Technique Taxonomy v1; BFO, Breaking Free Online; CAT, computer-assisted therapy; CBT, cognitive behavioral therapy; CONSORT, Consolidated Standards of Reporting Trials; ITEP, International Treatment Effectiveness Project; LBM, Lifestyle Balance Model; MET, motivational enhancement therapy; MRC, Medical Research Council; SUD, substance use disorder
